# Flexible Hybrid and Single-Component Aerogels: Synthesis, Characterization, and Applications

**DOI:** 10.1021/acs.langmuir.3c01811

**Published:** 2023-11-13

**Authors:** Mateusz Fijalkowski, Radek Coufal, Azam Ali, Kinga Adach, Stanislav Petrik, Huaitian Bu, Christian W. Karl

**Affiliations:** † Department of Advanced Materials, Institute for Nanomaterials, Advanced Technologies and Innovation (CXI), Technical University of Liberec, 461 17 Liberec, Czech Republic; ‡ Department of Science and Research, Faculty of Health Studies, Technical University of Liberec, 461 17 Liberec, Czech Republic; § Department of Material Sciences, Technical University of Liberec, 461 17 Liberec, Czech Republic; ∥ Department of Materials and Nanotechnology, SINTEF Industry, Forskningsveien 1, 0373 Oslo, Norway

## Abstract

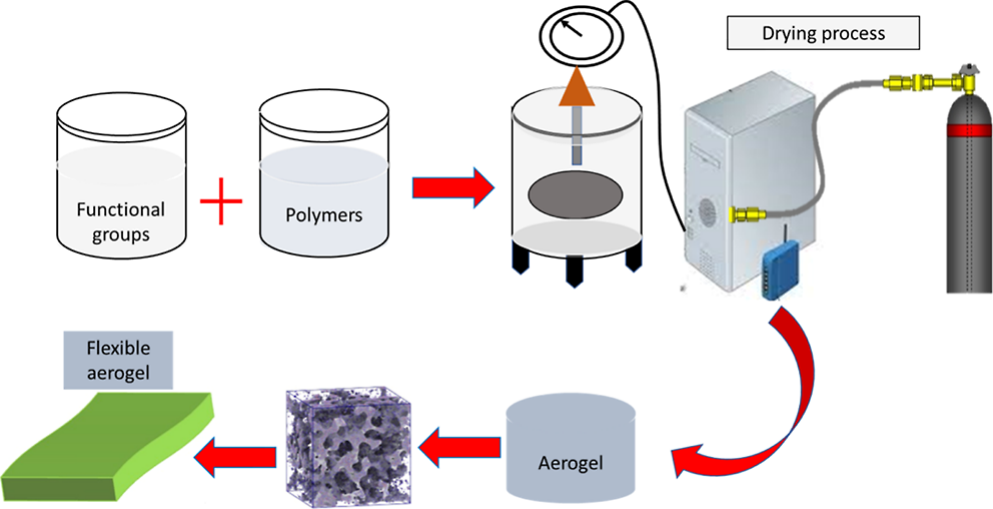

The inherent disadvantages of traditional nonflexible aerogels, such as high fragility and moisture sensitivity, severely restrict their applications. To address these issues, different techniques have been used to incorporate the flexibility in aerogel materials; hence, the term “flexible aerogels” was introduced. In the case of introducing flexibility, the organic part is induced with the inorganic part (flexible hybrid aerogels). Additionally, some more modern research is also available in the fabrication of hybrid flexible aerogels (based on organic–organic), the combination of two organic polymers. Moreover, a new type (single-component flexible aerogels) are quite a new category composed of only single materials; this category is very limited, charming to make the flexible aerogels pure from single polymers. The present review is composed of modern techniques and studies available to fabricate hybrid and single-component flexible aerogels. Their synthesis, factors affecting their parameters, and limitations associated with them are explained deeply. Moreover, a comparative analysis of drying methods and their effectiveness in the development of structures are described in detail. The further sections explain their properties and characterization methods. Eventually, their applications in a variety of multifunctional fields are covered. This article will support to introduce the roadmap pointing to a future direction in the production of the single-component flexible aerogel materials and their applications.

## Introduction

Aerogels are typically derived from wet gels, themselves prepared by sol–gel processes, and are dried using supercritical fluids, most often, CO_2_, freeze-drying, or evaporative drying. A wide variety of materials, including polymers, biopolymers, and metal oxides can be turned into aerogels.^1^ However, their complicated fabrication, high fragility, and brittle structure greatly limit their practical applications. To address the issue of brittleness and rigidity, different techniques have been used to incorporate the flexibility in aerogel materials.^2^ Hence, the term “flexible aerogels” was introduced. To date, the flexible aerogels can be categorized as “hybrid flexible aerogels” and “single-component flexible aerogels”.^3^ In the case of introducing flexibility by the formation of organic–inorganic structures (such as silica and organic part of polymers), the prepared aerogels are known as “hybrid flexible aerogels”. In such a system, Si–O–Si contributes to the inorganic part, and the Si–C bond represents the organic part,^4^ which creates flexible hybrid aerogels that have two components: one is inorganic and the other is organic. The inorganic part of the aerogel is brittle, while the organic part induced the flexibility to the structure due to the relatively more flexible organic chains.^3^

Furthermore, recent research approaches are also available for the fabrication of hybrid aerogels consisting of two organic polymers. Such aerogels can be denoted as organic flexible hybrid aerogels. Studies to develop organic and carbon aerogels were performed on different systems such as phenolic–furfural,^5^ cresol–formaldehyde,^6^ poly(vinyl chloride) (PVC) with 1,8-diazabicyclo[5,4,0]undec-7-ene (DBU),^7^ resorcinol–formaldehyde at ambient pressure,^8^ resorcinol–furfural,^9^ polyurethane,^10^ and phenol–furfural.^11^ An organic aerogel from hemicellulose citrate and chitosan was also developed by Salam et al.^12^

The second type, single-component flexible aerogels, are quite a new category composed of single materials that naturally possess mechanical flexibility at a given temperature without the need to be modified by other components. This category is very limited and is charming area for researchers to make the flexible aerogels pure from single polymers instead of the commonly used (inorganic–inorganic) or (organic–organic) hybrid system.^13^

Since the past decade, many studies have been published on the subject of (silica-based) rigid aerogels and hybrid flexible aerogels but not a single review available to differentiate between the composition of flexible hybrid and flexible single-component aerogels. The current review article aims to provide recent breakthroughs in the field of flexible hybrid and flexible single-component aerogels. We intend to assess the current technologies for the production of flexible hybrid and single-component aerogels, factors affecting their flexibility, along with their limitations and different drying methods (supercritical drying, freeze-drying, and ambient pressure drying). In the further sections, the properties and characterization methods of flexible aerogels are explained. Eventually, their applications in a variety of multifunctional fields are covered. This article will support to introduce the roadmap pointing to a future direction in the production of the single-component flexible aerogel materials and their applications.

## Preparation Methods for Flexible Aerogel Materials

### Flexible Hybrid Aerogel

The fragile nature of silica is still an unavoidable barrier in attempts to achieve a high mechanical strength of aerogels. The silica-based aerogels are brittle in nature, which constrains their large-scale application. The most promising way to overcome this drawback is the formation of an organic–inorganic hybrid aerogel. In such a system, Si–O–Si contributes to the inorganic part, and the Si–C bond represents the organic part; this combination brings about an improved hydrophobicity for aerogels and increases their mechanical strength.^3^ Hence, organic–inorganic hybrid aerogels are useful in special applications such as insulation at −40 °C for space exploration. Nowadays, elastic and flexible aerogels are prepared using different strategies such as cross-linking polymers with organosilane compounds, surface modification, and using bridging alkoxysilane precursors.^3,14^ The lack of mechanical flexibility in inorganic aerogels has led to the discovery of their organic counterparts. Even though the first organic aerogel was produced from cellulose 90 years ago, it was not until a few decades earlier that organic aerogel gained attention due to their flexibility, sustainable nature of the precursors, and tunable surface functionalities.^15^ So far, a wide variety of organic aerogels have been produced from various precursors, including synthetic polymers and biopolymers.^16^ Cellulose, alginate, chitosan, pectin, and starch are the most important biopolymers used for the fabrication of biopolymer aerogels.^15,17^

### Synthesis Methods of Flexible Hybrid Aerogels

**Figure 1:**
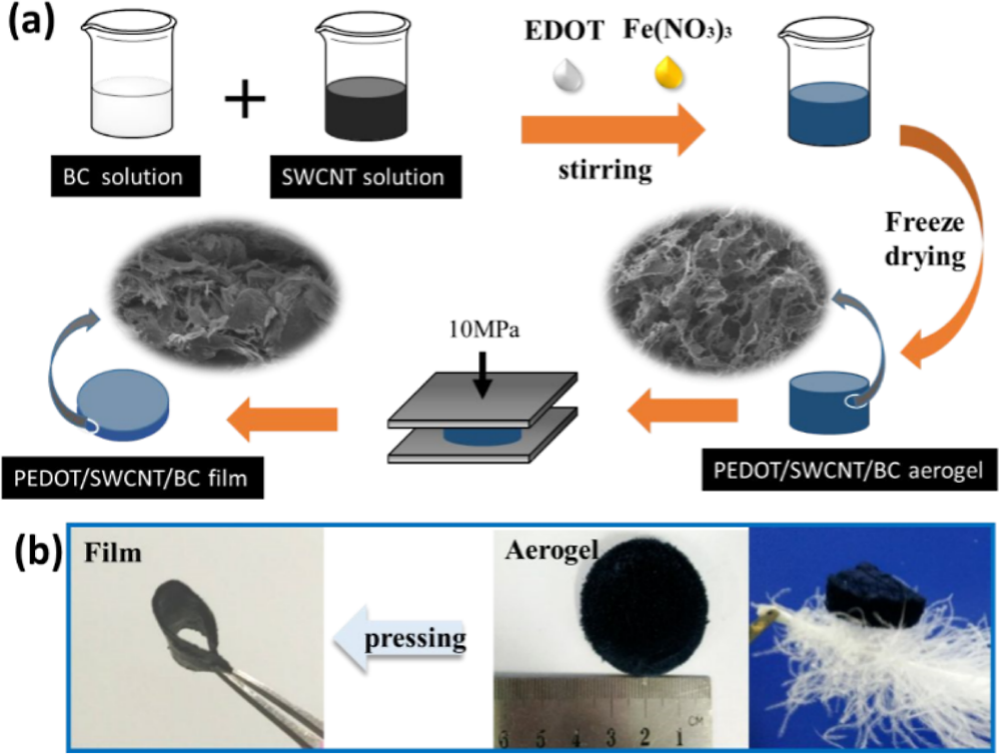
(a) Schematics showing the synthesis method for aerogels and flexible and porous PEDOT/SWCNT/BC films from aerogels. (b) Digital images of the PEDOT/SWCNT/BC aerogel and films as well as the lightweight PEDOT/SWCNT/BC aerogel placed on a fluffy feather. Reprinted with permission from Fang Jia, High thermoelectric and flexible PEDOT/SWCNT/BC nanoporous films derived from aerogels, published by ACS, 2019.^19^

The most important factor in deciding the stability and performance of hybrid materials is how different components integrate during the fabrication process.^18^ Therefore, the preparation of hybrid aerogels takes on two main routes: (i) physical and (ii) chemical integration.^15^ In the physical integration approach, the functional components are incorporated into the precursor (biopolymer or synthetic polymers) through physical entanglements, hydrogen bonding, or van der Waals interactions. However, the chemical integration approach involves the synthesis of functional components in the presence of precursor materials, which results in the development of stronger interactions via chemical bonding between the functional components and the polymer.^15^ Jia et al.^19^ synthesized flexible hybrid aerogels through a chemical method by adding Fe^3+^ and 3,4-ethylenedioxythiophene EDOT to the mixed solution of bacterial cellulose BC and single-walled carbon nanotubes (SWCNT), followed by the freeze-drying process. They further developed nanoporous films from hybrid aerogels with excellent electrical conductivity and low thermal conductivity by combining PEDOT, SWCNT, and BC in a hybrid mixture. Excellent electrical conductivity of 290.6 S/cm and low thermal conductivity of 0.13 W m^–1^ K^–1^ have been attained for a free-standing PEDOT/SWCNT/BC-32% film at room temperature through this technique. The prepared aerogels and films also exhibited exceptional flexibility, a fracture strength of 1.6 MPa, and a break elongation of 2.13% (Figure 1).^19^

**Figure 2:**
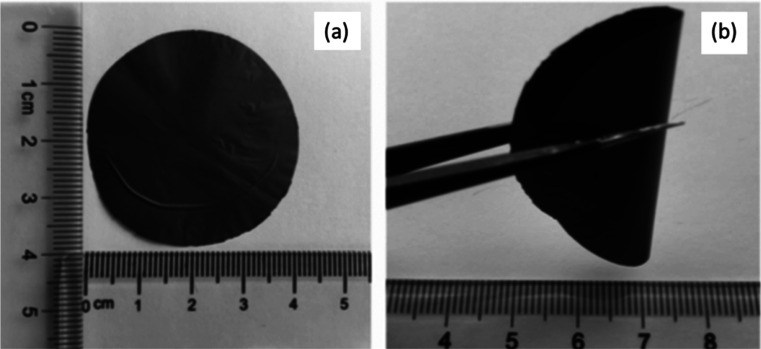
Figure shows the physical appearance of the hybrid aerogel (a) before twisting and (b) after twisting. Reprinted with permission from Miao Liu, Flexible MXene/rGO/CuO hybrid aerogels for high performance acetone sensing at room temperature, published by ELSEVIER, 2021.^20^

In another study, Liu et al.^20^ reported an effective method for the manufacture of ultrafine CuO nanoparticle-decorated three-dimensional (3D) flexible hybrid aerogel by using reduced graphene oxide (rGO) nanosheets and MXene (Ti_3_C_2_T_*x*_) as cobuilding blocks. The obtained 3D MXene/rGO/CuO aerogel exhibits strong acetone-sensing capability at ambient temperature attributed to the synergistic interactions of highly interconnected porosity networks, large targeted surface areas, homogeneous CuO NP dispersion, and high electron conductivity. The developed hybrid flexible aerogels exhibited exceptional flexibility as shown in Figure 2.^20^

**Figure 3:**
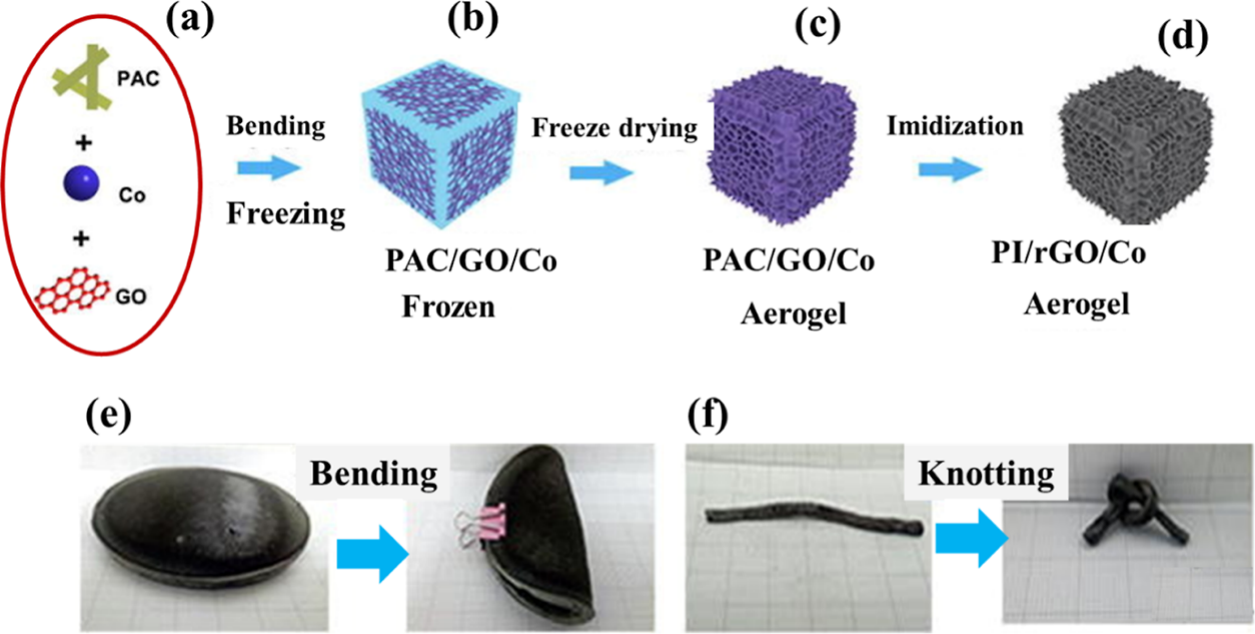
(a) Schematics showing the synthesis of aerogels, (b) images showing the flexibility of synthesizing aerogels. (c) PAC/GO/Co aerogel, which was the prototype for the PI/rGO/Co aerogel. (d) Final PI/rGO/Co aerogel, which was obtained after imidization at 250 °C in a nitrogen atmosphere, (e) digital images showing the excellent flexibilities during bending, and (f) during knotting of the variform PI/rGO/Co aerogels. Reprinted with permission from Xinhai Zhang, Double-cross-linking strategy for preparing flexible, robust, and multifunctional polyimide aerogel, published by ELSEVIER, 2020.^23^

Cellulose nanocrystal (CNC) aerogels are naturally brittle due to the stiffness of the crystals and the restricted mobility of the individual crystals. Conventionally, low molecular mass cross-linkers have been used to enhance the toughness property of aerogels; however, little attention has been paid to improve the flexibility of aerogels. In this context, Zhou et al.^21^ paved the path for the development of nanomaterial-based aerogels having excellent mechanical properties and functionality through the incorporation of functional polymers using the click chemistry reaction. In this work, the CNC–PCL hybrid aerogel with improved toughness and flexibility was synthesized using a macromolecule-based click cross-linking technique. The polycaprolactone diol (PCL) was used as a cross-linking agent.^21^ Rezaei et al.^22^ also presented a novel method for the production of a flexible, thermally superinsulating hybrid silica aerogel. They introduced a fast method to fabricate a polyether-based hybrid silica aerogel with less gelation time. The preparation time for wet gels, which includes gelation, aging, and solvent exchange, was cut from many days to just a few seconds with this novel technique. The resulting aerogels exhibited excellent mechanical and flexible properties, as well as thermal superinsulation. Likewise, Zhang et al.^23^ reported an innovative double-cross-linking technique to develop a freeze-dried, durable polyimide/reduced graphene oxide/cobalt (PI/rGO/Co) aerogel with an interdigitated cellular architecture. The graphene oxide (GOsheets) hydrogen bonds and the Co ion coordination interactions were believed to be responsible for the double-cross-linking method. The interdigitated cellular architecture was a crucial component in order to produce aerogels with low densities, good fatigue resistances (1000 cycles), flexibility, and strong moduli (0.506 MPa compression modulus and 43% increase in tensile modulus). The developed aerogels were highly flexible as shown in Figure 3.^23^

### Parameters Affect the Flexibility of Hybrid Aerogels

The flexibility of hybrid aerogels is greatly affected by two main factors, i.e., the concentration of the hydrogel and the molar ratio of the organic polymer to the cross-linking agent. According to a study, it was established that the flexibility of the hybrid aerogel is increased when the molar ratio of the cross-linking agent is higher than that of the organic polymer. It was also concluded that the flexibility of aerogels is also increased by increasing the concentration of hydrogels.^24^

### Limitations in Flexible Hybrid Aerogel Materials

The compression strength and flexibility of aerogels can both be increased by the addition of polymers such as PMMA, xylan, PLA, and cellulose ester onto the framework of the aerogels. Such hybrid aerogels present some flexibility due to chain mobility of the polymers and durability due to the chain entanglement among polymeric structures. The dimensional stability and chemical stability of aerogels are, however, decreased due to the generally low interfacial interaction between polymers and the skeleton of the aerogel. Furthermore, the architectural bonding between the components is not substantially altered by this polymer blending technique.^25^ Therefore, a significant increase in the concentration of polymers is typically needed to enhance the mechanical properties of hybrid aerogels.^26^ The choice of flexible macromolecules with a controlled molecular weight for joining the organic polymers is the other limitation of state-of-the-art studies. The degree of macromolecule cross-linking on the surface of organic polymers is significantly reduced by the grafting difficulty of macromolecule cross-linking, which is driven by the limited reactive ends offered by macromolecules and the significant steric hindrances.^27^

### Polymeric Aerogels–Single-Component Aerogels with Inherent Flexibility

Aerogels most commonly are based on silica.^28^ Despite its record-low thermal conductivity, the fragility and brittleness of silica aerogel need to be improved for applications where mechanical strengths are important. Recent progress has been made in developing aerogels involving organic or nanostructured components to improve the mechanical properties.^29^ On the other hand, they can also define the “inherently flexible aerogels” as those aerogels composed of single materials that naturally possess mechanical flexibility at a given temperature without the need to be modified by other components. However, this category is very limited (only few studies available), and most of the available single-component flexible aerogels can be “technically” included in this group.^30^

### Synthesis of Single-Component Aerogels: Methods and Schematics

**Figure 4:**
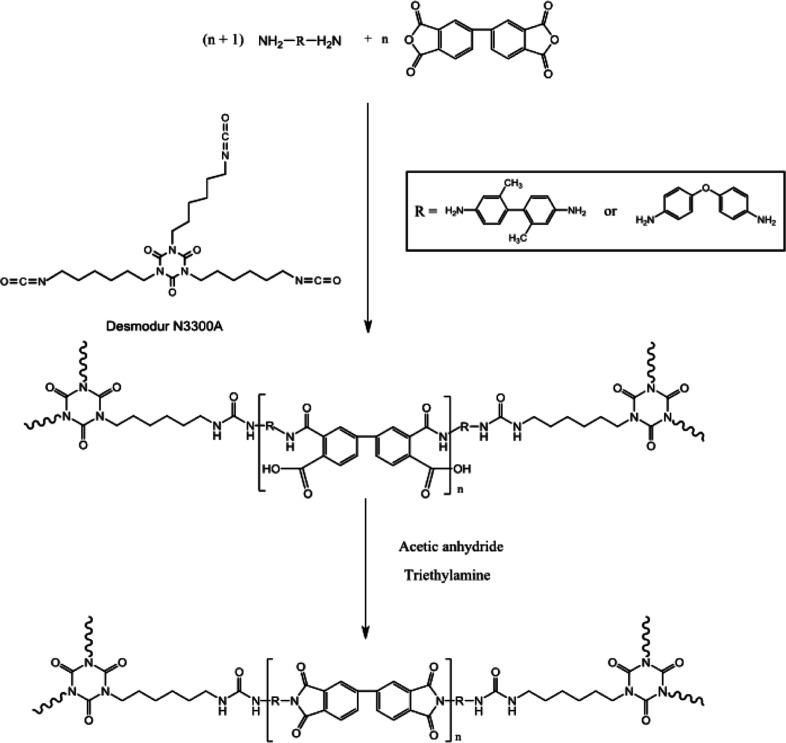
Schematic illustration of the synthesis of single-component aerogels. Reprinted with permission from Baochau N. Nguyen, Polyimide aerogels using triisocyanate as cross-linker, published by ACS, 2017.^31^

In recent years, scientists have focused on making aerogels from organic polymers instead of materials such as silica and metal oxides. Some examples of organic polymer-based aerogels include those made of cellulose, polyurethane, melamine/formaldehyde, and resorcinol/formaldehyde.^28,30^ However, more recently, it has been revealed that aerogels produced from a single engineered polymeric component, such as PI or polyamide, have superior mechanical performance compared to organic-based hybrid aerogels. Nguyen et al.^31^ conducted a study to develop a variety of PI-based aerogels by using an economical tri-isocyanate-based Desmodur N3300A cross-linker. In fact, these aerogels were fabricated by using the tri-isocyanate cross-linker to cap the links within amine-terminated PI oligomers. 3,3′,4,4′-Biphenyltetracarboxylic dianhydride (BPDA), along with diamine components such as 2,2′-dimethylbenzidine (DMBZ) and 4,4′-oxidianiline (ODA), or a combination of both were used to produce the PI oligomers. Subsequently, the formed PI oligomers were chemically imidized at ambient temperature. Generally, different kinds of polyfunctional amines are incorporated as cross-linkers to produce PI aerogels. The amines included 1,3,5-tris(4-aminophenoxy)benzene (TAB), 1,3,5-tris(aminophenyl)benzene (TAPB), 2,4,6-tris(4-aminophenyl) pyridine (TAPP), and octa(aminophenoxy)silsesquioxane (OAPS). The schematic representing the step-by-step formation of aerogels starting from raw materials to end invention is shown in Figure 4.^31^

**Figure 5:**
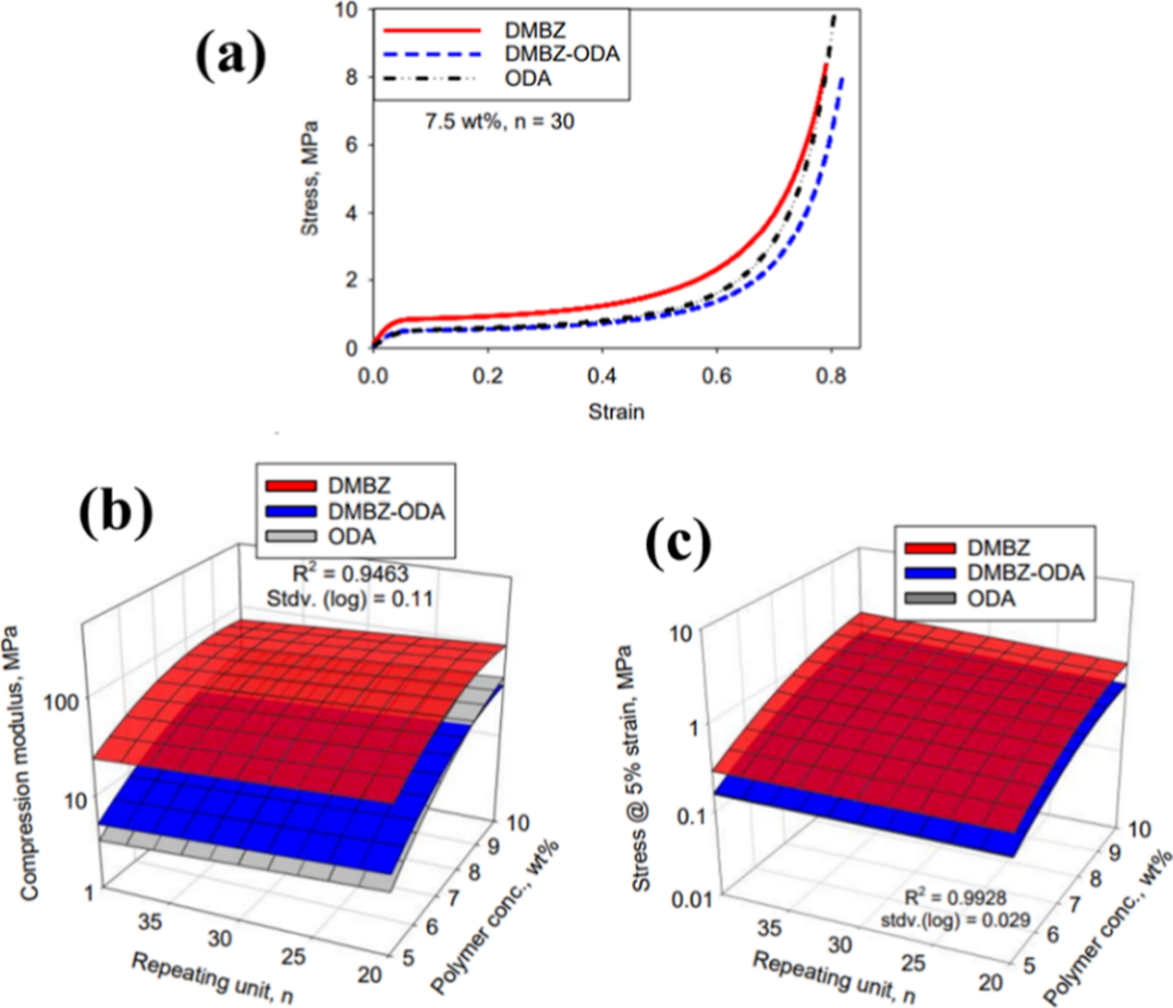
Graphs of (a) typical stress–strain curves for compression of PI at 7.5 wt % solid and empirical models for (b) compression modulus vs polymer concentration strength and (c) stress vs polymer concentration and n. Reprinted with permission from Baochau, Polyimide aerogels using tri-isocyanate as cross-linker, published by ACS, 2017.^34^

The tensile and compression analyses of the as-prepared aerogels were conducted on dog bone and cylindrical shaped samples, respectively. The compression stress–strain curves for the PI aerogels composed of 100 mol % DMBZ, 50 mol % DMBZ, as well as 50 mol % ODA and 100 mol % ODA with 7.5 wt % solid are illustrated in Figure 5a. The compressive modulus and strength have been reported to be reliant on the flexibility or rigidity of the polymer backbone in several research studies depending on other cross-linkers.^32,33^Figure 5b,c indicates that despite possessing a lower density, aerogels produced with 100 mol % DMBZ exhibited the highest values for Young’s modulus.^33^

**Figure 6:**
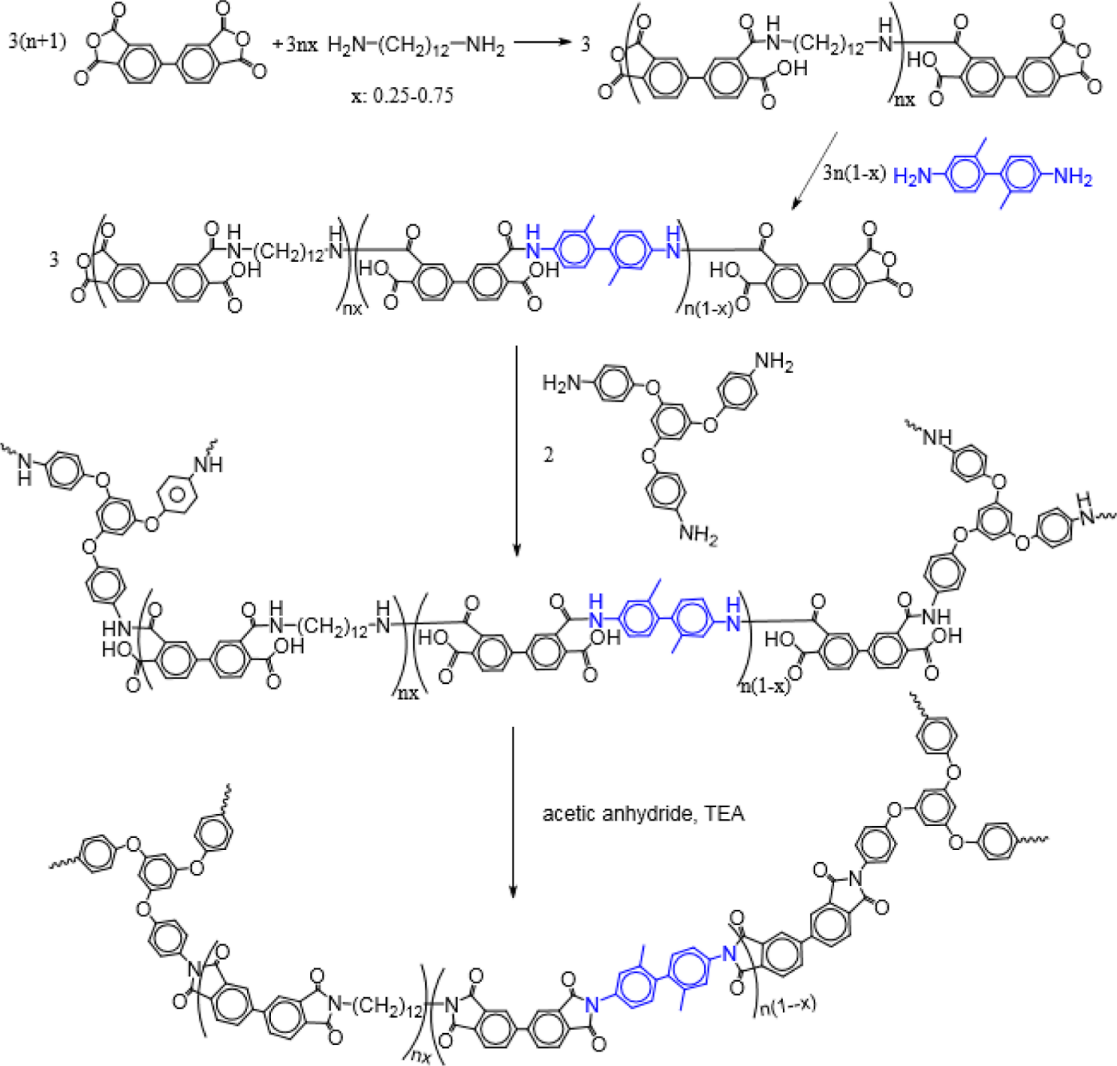
Schematic illustration of the synthesis route of aerogels from DADD and DMBZ. Reprinted with permission from Haiquan Guo, Flexible polyimide aerogels with dodecane links in the backbone structure, published by ACS, 2020.^35^

Guo et al.^35^ reported the synthesis of PI aerogels using 1,12-dodecyldiamine (DADD), 3,3′-dimethylbenzidine (DMBZ), and 3,3′,4,4′-biphenyltetracarboxylic dianhydride (BPDA) and cross-linking with 1,3,5-triaminophenoxybenzene (TAB). The schematic illustration of the synthesis route of the aerogel from the raw material is shown in Figure 6. The produced aerogel was more flexible in its backbone structure when the aromatic diamine, DMBZ, was substituted with various concentrations of the aliphatic diamine, DADD.^35^

**Figure 7:**
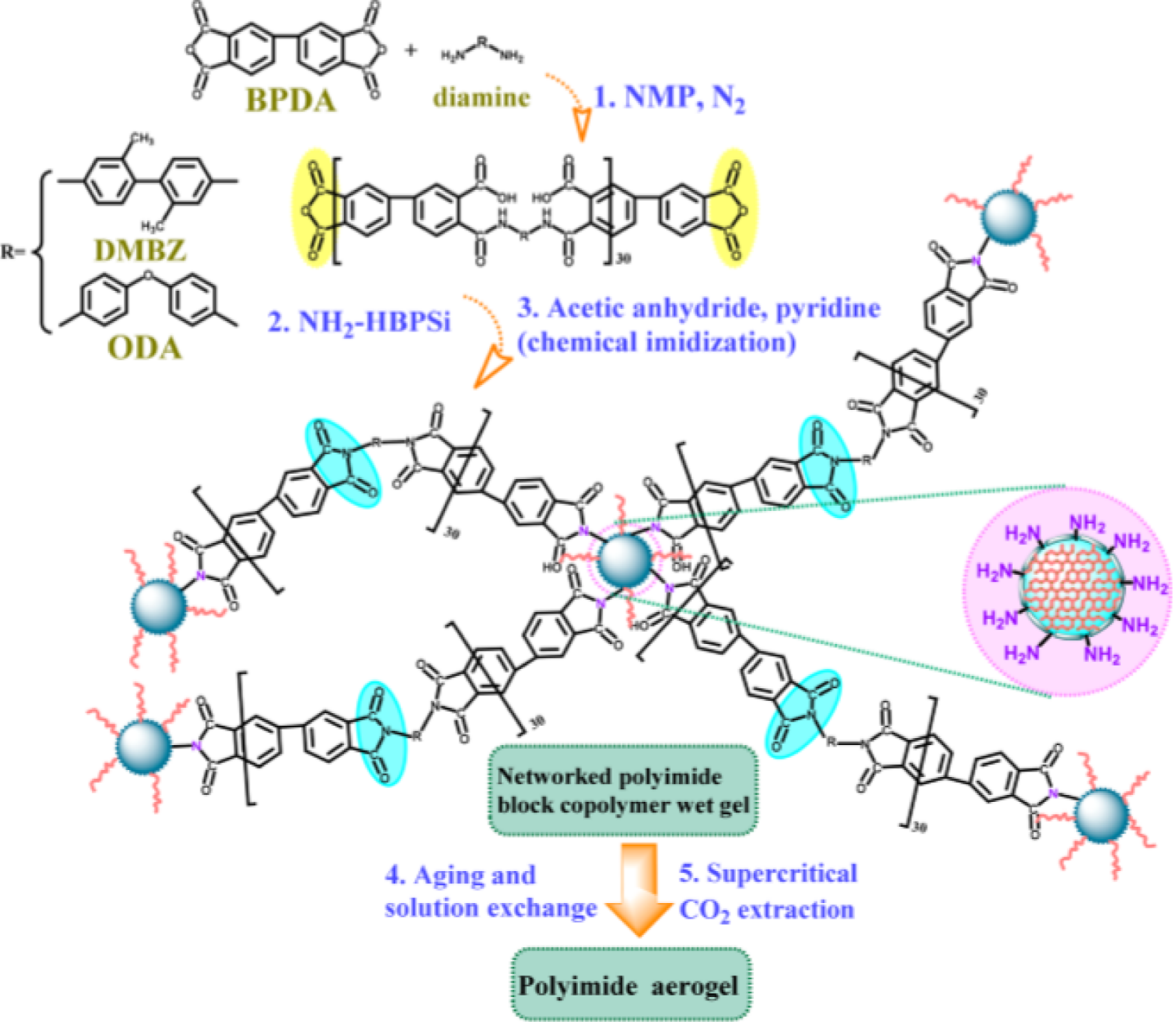
Schematic illustration of the PI aerogel synthesis from raw materials. Reprinted with permission from Tingting Wu and Jie Dong, Fabrication of polyimide aerogels cross-linked by a cost-effective amine-functionalized hyperbranched polysiloxane (NH_2_–HBPSi)], published by ACS, 2020.^36^

In another study, Wu et al.^36^ developed PI aerogels using polyamic acid (PAA) oligomers with anhydride end-caps that were then connected together by a cheap, lab-made, amine-functionalized hyperbranched polysiloxane macromer (NH_2_–HBPSi). Phenyltrimethoxysilane, tetraethoxysilane, and γ-aminopropylmethyl diethoxysilane silane coupling agents were combined to form NH_2_–HBPSi in a hydrolysis–condensation reaction (Figure 7). The resulting PI aerogels showed similar properties to the triamine cross-linked PI aerogels in terms of high BET-specific surface areas, low densities, low thermal conductivity, and excellent mechanical and thermal properties over the entire range of NH_2_–HBPSi cross-linker concentrations used, i.e., 2.5–12.5 wt %.^36^

**Figure 8:**
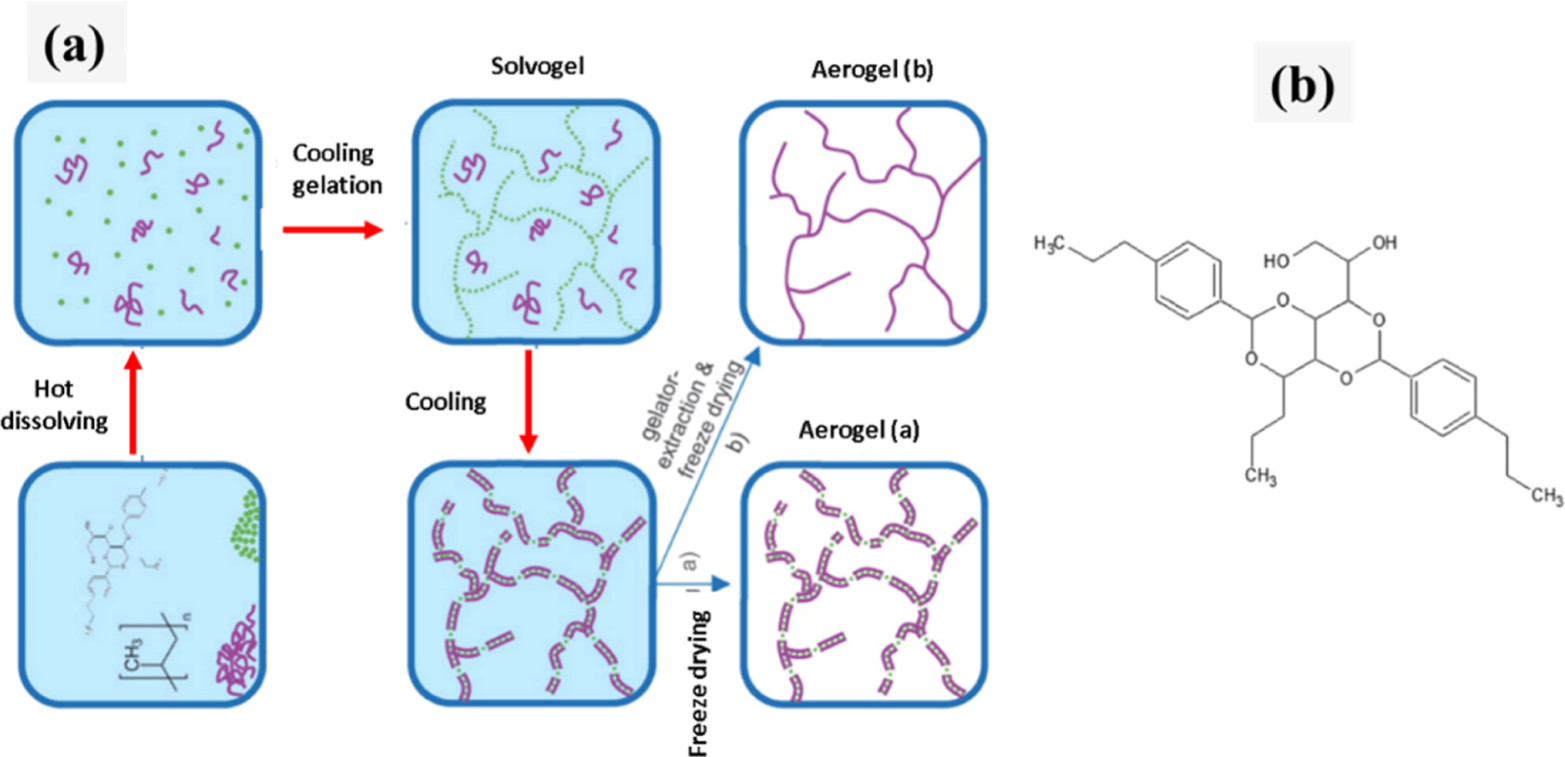
Schematic overview of the (a) production of organogelator-based aerogels (polymer–purple/gelator–green and (b) 1,2,3-trideoxy-4,6:5,7-bis-*O*-[(4-propylphenyl)methylene]-nonitol (TBPMN). Reprinted with permission from Felix, Novel finely structured polymer aerogels using organogelators as a structure-directing component, published by RSC, 2021.^37^

Leven et al.^37^ prepared polyolefin-based aerogels by using freeze-drying. For the fabrication of the aerogel (Figure 8a), predetermined amounts of the gelator (TBPMN; Figure 8b) and polymer (PE or PP) were added to the high-boiling solvent trichlorobenzene (TCB or another solvent with comparable phase compatibility) and heated under continuous stirring to produce a homogeneous solution. The solution was cooled down with around 5 K min^–1^ after the agitation stopped. Low-concentration gelation (1.5% gelator/polymer solution and below) was resulted in a bluish color. Polymer crystallization was followed by increased turbidity or even a white color, particularly at high polymer concentration. The gel body was removed out of the container, and the solvent was replaced by repeatedly washing it (a minimum of three times) with benzene diluted 4 times of its actual concentration. After that, freeze-drying was done at 0 °C under 3 mbar high vacuum.

The formerly translucent gel bodies transformed into opaque, white aerogels with a relatively small volume contraction. The extraction solvent was cooled to separate into two phases in order to recover the gelator. A separating funnel was used for the removal of TCB. The resulting aerogels have remarkable mechanical stability, fine-grained structures, and poor thermal conductivity.^37^ A mechanism for the development of structure was hypothesized after studying the effects of the type of polymer, molecular weight, concentration on structure, thermal conductivity, and compressive strength.

### Parameters Affect the Flexibility of Single-Component Aerogels

The backbone structure of PI oligomers plays a greater role in PI aerogels than does the cross-linker. A different backbone chemistry has been demonstrated to cause variations in processing shrinkage, which results in fluctuations in density and other qualities that depend on density, including modulus and moisture retention. Density can also be altered in PI aerogels with the same chemistry at the backbone by adjusting the concentration of the precursor solution.^38^ In addition, the flexibility of single-polymer-based aerogels is also influenced by the specific synthesis methods employed during their fabrication. The aerogels produced by sol–gel polymerization showed the variation in flexibility based on polymerization conditions and cross-linking agents.^39^ Supercritical drying preserves the polymer network connectivity and decreases the capillary forces to avoid the structural collapse. Alternatively, the solvent evaporation (from the gel under atmospheric pressure) with ambient-pressure-drying method can lead to structural collapse.^40^ During the freeze-drying process, the choice of monomers and sublimating conditions of solvents influence the flexibility of the resulting polymer aerogel.^41^ Furthermore, the incorporation of some additives such as flexible polymers during the synthesis of aerogels results in a soft component and flexible structures. Moreover, some certain parameters such as temperature, production time, blending of cross-linkers, and surface modifications can be selected in accordance to the desired application and specific properties, including flexibility.^42^

### Limitations in Flexible Single-Component Aerogels

Although the polyamide (PI) aerogels have drawn attention from all around the world, their practical applications are still limited. This is probably due to the reason that cross-linkers are expensive and commercially unavailable, which prevents them from being scaled up.^43^ There has been a heck of a lot of preliminary research on the formation of PI aerogels using polyamic acid oligomers with amine or anhydride end-capped that are cross-linked using multiamine cross-linkers such as 1,3,5-benzenetricarbonyl trichloride (BTC), 1,3,5-triaminophenoxybenzene (TAB), octa(aminophenoxy)silsesquioxane (OAPS), or poly(isobutylene-alt-maleic anhydride) (PMA-D).^44,45^ All these cross-linkers are relatively expensive.^46^ Some studies have explored using chemically modified nanoparticles as cross-linkers, such as silver nanoparticles, carbon nanotubes, and octa(aminophenyl)silsesquioxane (OAPS), to produce PI aerogels. These chemically processed nanoparticle cross-linking agents are, however, not commercially available. Also, the synthesis methods involve potentially hazardous processes.^47^ These are the two key factors which curtails the large-scale synthesis of single-component PI aerogels.^48^

### Drying Methods

It is widely known that drying methods have a significant impact on the surface area and porosity of the resulting aerogel.^49^ The drying process is the final and most important phase in the fabrication of aerogel. A wet gel formed by polysaccharide polymers has a heterogeneous structure and a high porosity level filled with water. The purpose of the drying process is to eliminate the liquid that is trapped inside the pores. It is well known that conventional drying methods lead to the development of capillary tensions when the vapor–liquid interface recedes into the porous structure, causing the material to shrink and eventually disintegrate.^50^ Thus, the goal of employing advanced drying techniques like supercritical drying (CO_2_, methanol, acetone, or ethanol), freeze-drying, and ambient pressure drying is to remove the solvent presenting inside the pores of wet-pore gel, while sustain the structure’s high degree of porosity and prevent the aerogel volume from breaking down, disintegrating, or collapsing. The most cutting-edge drying methods for wet gel processing are listed in the following section.^51^

### Freeze-Drying Method

Freeze-drying is an additional drying method used to protect the porosity of the network. This technique involves cooling the pore liquid to its freezing temperature first and then sublimating the frozen solid under vacuum to remove it without damaging the pore walls. The porous structure developed through this approach is known as cryogels, and they have a surface area and pore volume that are relatively smaller than the aerogels.^52^ Similar to supercritical drying, the pore liquid should be replaced with a different liquid that has a low freezing point and expansion coefficient.^41^ However, the 3D network structure collapses as the pore liquids are crystallized inside the pores.

### Ambient Pressure Drying Method

The freeze-drying and supercritical drying methods are not practical techniques for the commercial manufacturing of aerogels. However, an ambient pressure drying method is facile and practically economical for the large-scale manufacture of aerogels.^53^ As a result, several techniques were used to drain pore fluids in an ambient environment without causing the network to shrink. In fact, the contact angle between the pore walls and pour liquid normally increased by the addition of surfactants in order to reduce the capillary forces acting on the pore walls. The surface hydroxyl groups (–OH) on both outer and inner surfaces of the network structure were altered with bulky groups including chlorotrimethylsilane or hexamethyldisiloxane.^54^ As a result, the surface of the gel network turns extremely hydrophobic, and its reactivity is quite low. The gel network is then dried using the ambient pressure drying method. The gel network is contracted when the pore liquid evaporates. However, the bulky groups inhibit the cross-linking processes (Si–O–Si and Ti–O–Ti) between the surface hydroxyl groups (–OH), rendering the shrinkage reversible. As a result, the network enlarges again back to its original size, a phenomenon known as the “spring back effect”. Additionally, a highly viable solvent-exchange technique was used to generate an aerogel nanostructure in ambient environments. This method successfully replaced the pore liquid with a liquid having low surface tension without damaging the walls of the pores in the aerogel structure.^55^

### Supercritical Drying Method

**Figure 9:**
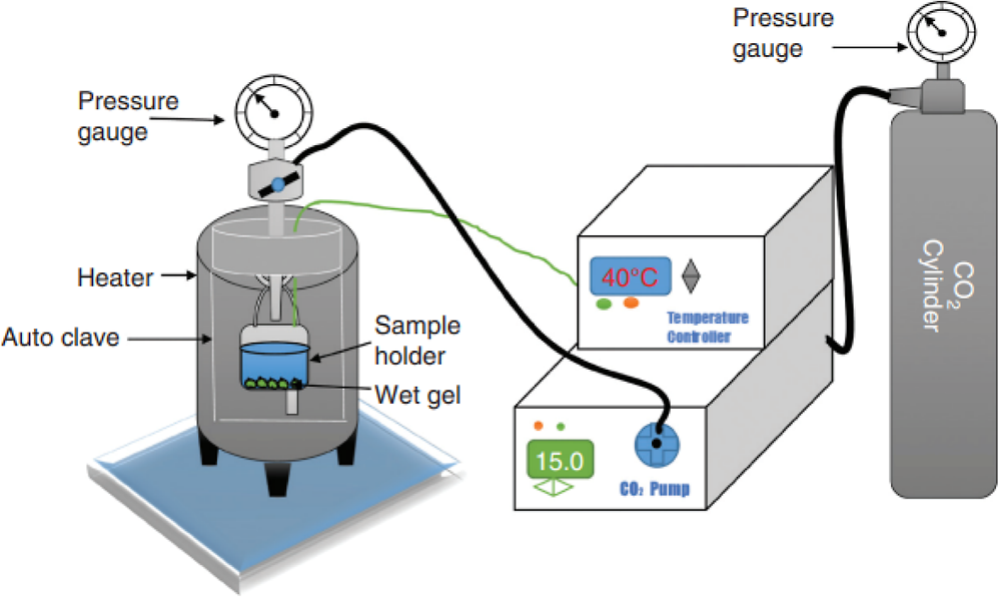
Schematic illustration of the supercritical drying setup. Reprinted with permission from Mehrez E. El-Naggar, Synthesis, drying process, and medical application of polysaccharide-based aerogels, published by ELSEVIER, 2019.^58^

Supercritical drying, as depicted in Figure 9, is an alternate drying method to conventional drying that can create an aerogel while maintaining porosity. It also maintains the remarkable textural features of the wet gel in solid form (drying state) and prevents the pores from collapsing.^56^ In practice, traditional supercritical drying entails heating wet gel in a closed container to a temperature and pressure that are higher than the critical values for the solvent that is trapped in the pores of the gel. Vapor phase and liquid phase get indistinguishable like supercritical fluids.^57^ Additionally, the venting process does not include capillary forces. The aerogel can be removed from the autoclave once the fluid has been released through the outlet valve and cooled subsequently. Enough solvent must be delivered during the whole drying process in order to prevent the aerogel solid from shrinking and breaking as well as to guarantee the reliability of supercritical conditions.^58^

There are two types of supercritical drying techniques: a low-temperature supercritical drying method and a high-temperature supercritical drying method. The organic solvents in gel, such as acetone, ethanol, and methanol, can be replaced by employing soluble CO_2_ in a low-temperature supercritical drying method which is followed by its transformation into supercritical carbon dioxide (Sc-CO_2_).^59^ The transformation takes place at a critical temperature, which is extremely close to room temperature, which then results in the development of aerogel. However, capillary pressure can be removed via supercritical drying, maintaining the natural shape of components. This technique has the advantage that surface tensions in pores can be avoided and, thus, it maintains the porous structure of aerogels. In the high-temperature supercritical drying technique, the hydrogel should be replaced with an organic solvent (acetone, ethanol, or methanol) before being placed in an autoclave for pressurization and heating. When the solvent achieves the supercritical stage, it vents from the gel.^60^ However, supercritical drying was the preferred method for the development of organic aerogels because of surface forces in the pores of various sizes during drying. This behavior precludes dimensional shrinkage as the capillary strains are no longer present at the supercritical levels. Additionally, CO_2_ supercritical drying was required to be used at mild supercritical temperature (*T* around 31 °C) to prevent the thermal degradation of organic components.^61^

### Effect of Drying Methods on the Structure of Porous Materials

**Figure 10:**
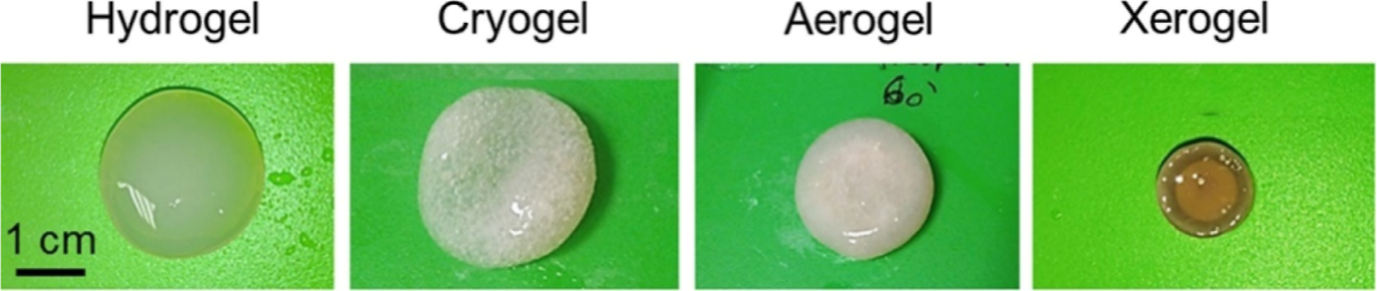
Examples of a pectin hydrogel, cryogel, aerogel practical samples of a pectin hydrogel, cryogel, aerogel, and xerogel. Reprinted with permission from Tayyab, Aerogels for biomedical, energy and sensing applications, published by MDPI, 2021.^62^

The drying techniques utilized for the porous materials, such as aerogels, gels, and foams, can have a significant impact on the final structure. Properties including porosity, mechanical strength, general morphology, and pore size distribution are significantly influenced by the drying techniques.^62^ The pore size distribution, mechanical stability, and porosity of porous materials are all impacted by the drying techniques. Supercritical drying frequently produces well-maintained structures with high porosity, whereas the conventional drying techniques might result in variable degrees of structural modifications in addition to reduced porosity. The precise material composition, intended function, and required structural characteristics must all be taken into consideration when choosing a drying technique. Research was conducted to analyze the impact of different drying methods on the release and structural properties of aerogels, cryogels, xerogels, and pectin hydrogels. They applied supercritical drying, freeze-drying, and evaporative drying under low vacuum to fabricate porous pectin aerogels, cryogels, and xerogels. They examined whether the freeze-drying technique resulted in low-density samples in contrast to pectin hydrogels and cryogels. Figure 10 depicts the test specimens with a dry core in pectin aerogels, confirming the slow solvent transport across the dry system.^63^

### Limitations of Drying Techniques

All the above-mentioned techniques are advantageous, but they have their own limitations. Freeze-drying is more preferred due to its widespread use and advantages over supercritical drying such as ease of application, cost-effectiveness, and suitability for the development of highly structured and ordered porous aerogels. Numerous aerogels can be prepared using this drying technique on an industrial scale.^64^ However, one of its disadvantages is the pollution that solvent evaporation causes to the environment, people, and animals. The supercritical drying system is an effective method for preventing drying shrinkage or mesopore collapse in order to obtain well-characterized structures. However, the use of high temperatures and pressures during the traditional supercritical drying process, the vast amounts of solvent used, and the time it takes for solvent exchange considerably reduce their probability of scaling up to an industrial scale. This restriction could be due to the overall high cost-increasing energy and time consumption of the process.^58^

## Properties of Flexible Aerogels

**Figure 11:**
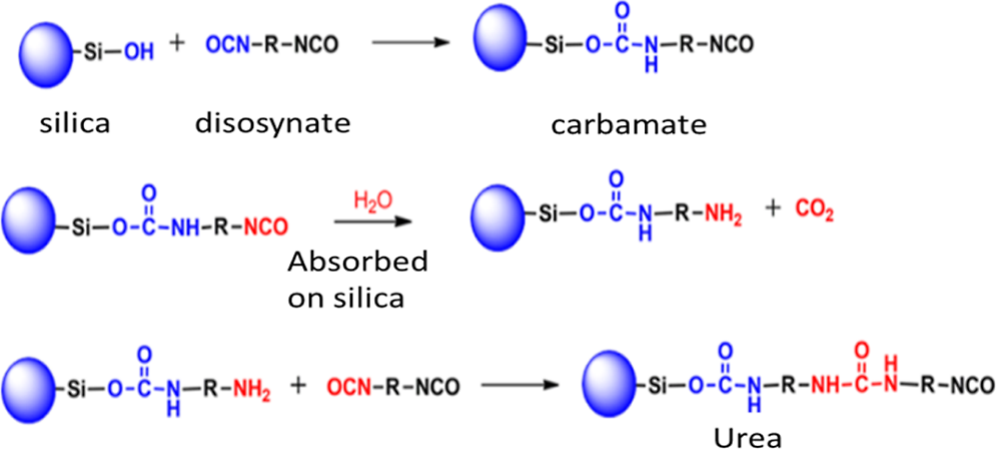
Mechanism for the cross-linking of silica with diisocyanato. Reprinted with permission from Nicholas Leventis, Three-dimensional core–shell superstructures: mechanically strong aerogels, published by ACS, 2007.^65^

Flexible low-density aerogels have received increasing attention due to their unique properties. Their microscopic geometry is a crucial factor to determining their mechanical functions, i.e., strength and toughness (flexibility). Many studies have reported the detailed investigations of chemical, mechanical, physical, and morphological properties of aerogels. Leventis^65^ prepared the isocyanate cross-linked silica aerogels by dissolving the di- and tri-isocyanates into a silica network in the presence of isocyanate solution. The fabricated hybrid aerogels (silanols with isocyanate groups) were subjected to a three-point bending test. It was noted that hybrid aerogels were able to tolerate a higher load as compared to simple pure silica aerogels. In fact, the load bearing capacity was increased due to the cross-linking between an isocyanate and surface hydroxyl groups. The surface hydroxyl groups of silica yield an amine, which in turn reacts with excess isocyanate to form polyurea. As a result, polyurea is referred to as the cross-linked polymer made of isocyanate. Figure 11 illustrates the chemical reaction that occurs when silica cross-linked with urea.^65^

In a recent study, Churu et al. used an acid-catalyzed sol–gel process to create the isocyanate cross-linked aerogel. They introduce the triblock copolymer Pluronic P123 as a structure-directing agent to enhance the mechanical properties.^66^ This selected approach enhanced the strength (said young modulus almost 800 MPa), while the specific energy absorption (123 J/g) and low density render this material a good choice for ballistic protection. Kong et al. subjected the copolymerization of tetraethylorthosilicate (TEOS) with APTES in the presence of derived toluene diisocyanate, via ambient pressure drying.^67^ The authors noticed that the compressive strength did not significantly change by polymer incorporation, while there was a significant increase in the elastic modulus. Mandal et al. assembled transparent hybrid aerogels by varying the total silane and isocyanate concentrations in a TMOS-*co*-APTES system and cross-linking them with triisocyanate.^68^ The optimized aerogel exhibited a low bulk density (0.25–0.33 g/cm^3^), moderate porosity (76–83%), low thermal conductivity (18–22 mW/mK) and enhanced mechanical properties (Young’s modulus of 30–70 MPa). Table 1 demonstrates the different mechanical properties of the flexible aerogels.

**Table 1:** Overview of the Properties of Reported Flexible Aerogels

precursor	synthesis and drying technique	key properties	suitable applications	ref
methyltrimethoxysilane (MTMS)	two-step sol–gel process and SAD supercritical alcohol drying	flexible aerogels having compressibility ∼60%, with water contact angle ∼164°	insulation	(69)
			oil spill clean up	
TEOS and polyrotaxane	one-pot base-catalyzed sol–gel process and SCD	flexible and transparent aerogels, low thermal conductivity 0.012–0.015 W/m·K, density ∼0.15–0.30 g/cm^3^	medical applications	(70)
			building insulation	
			solar energy	
MTMS and DMDMS	two-step sol–gel process and SCD	highly flexible aerogels having spring back behavior stress (∼0.10 MPa)	protective gear	(71)
			flexible electronics	
MTMS	single-step sol–gel process and SCD supercritical CO_2_ drying	transparent and flexible aerogels with 80% linear compression and 95% recoverability	pressure relief mattresses	(72)
			medical devices	
MTESt	two-step sol–gel process and SAD	elastic, flexible, and superhydrophobic aerogels, Young’s modulus ∼3.95 × 10^4^ N/m^2^	structural components	(73)
methyl-triethoxysilane			impact absorption	
aromatic diamine	supercritical CO_2_ drying	flexible	flexible applications	(74)
triisocyanate with silane	supercritical CO_2_ drying	low bulk density (0.25–0.33 g/cm^3^), porosity (76–83%), Young’s modulus of 30–70 MPa	oil spill clean-up	(75)
			air filters	
			insulations	
MTES methyltriethoxysilane	two-step sol–gel process and SAD	light, flexible, hydrophobic, and oleophilic silica aerogels with water contact angle ∼157°	electronics protection	(76)
		maximal stress ∼15.09 kPa	marine equipment	
		portable shelters		
VTMS vinyltrimethoxysilane	two-step sol–gel process using surfactant and SCD supercritical CO_2_ drying	transparent and flexible aerogels with 50% compression and almost 100% resilience	vibration dampening	(77)
		thermal conductivity ∼0.0153 W/m·K	impact absorption	
MTMS and GPTMS	two-step sol–gel process and SCD	flexible aerogels with 100% recoverability, Young’s modulus ∼0.46 MPa	smart textile	(78)
		thermal conductivity ∼0.0336 W/m·K	sensors	
			flexible electrodes	
TEOS and aramid fibers	single-step sol–gel process and APD	flexible aerogels with thermal conductivity between 0.0221 and 0.0235 W/m·K	building insulation	(79)
			pipe cooling	

## Characterization Methods of Flexible Aerogels

Characterizing flexible aerogels involves a comprehensive examination of their physical, mechanical, and thermal properties to assess their suitability for specific applications. Thermal conductivity measurements help gauge their insulating capabilities, while porosity analysis offers information about internal structure and surface area. The strength of polymeric aerogels is normally measured by a tensile test method. Samples of aerogels in dog bone shape, having dimensions about 5.2 mm width and 4.9 mm thickness, are prepared for tensile testing according to ASTM D882. The ASTM standard D695-10 has been reported by many researchers to measure the compressibility of flexible insulating aerogels. Details about their elasticity, durability, and flexibility under various conditions were provided by three-point bending analysis. In accordance with ASTM D790-15, rectangular specimens measuring 10.2 mm long by 8.3 mm wide and nominally 1.8 mm thick were produced via a TA Instruments Q800 DMA (dynamic mechanical analysis). SEM also revealed the morphology of the surface, which was helpful in determining microstructural characteristics. X-ray diffraction (XRD) and Fourier transform infrared (FTIR) spectroscopy are examples of techniques that can be used to examine the molecular structure and chemical composition. The optimization of flexible aerogels for various applications, such as cushioning in electronics and thermal insulation in building, is determined by these characterizations collectively.^80^

### Effect of Pore Sizes and Pore Size Distribution on Mechanical Properties

**Figure 12:**
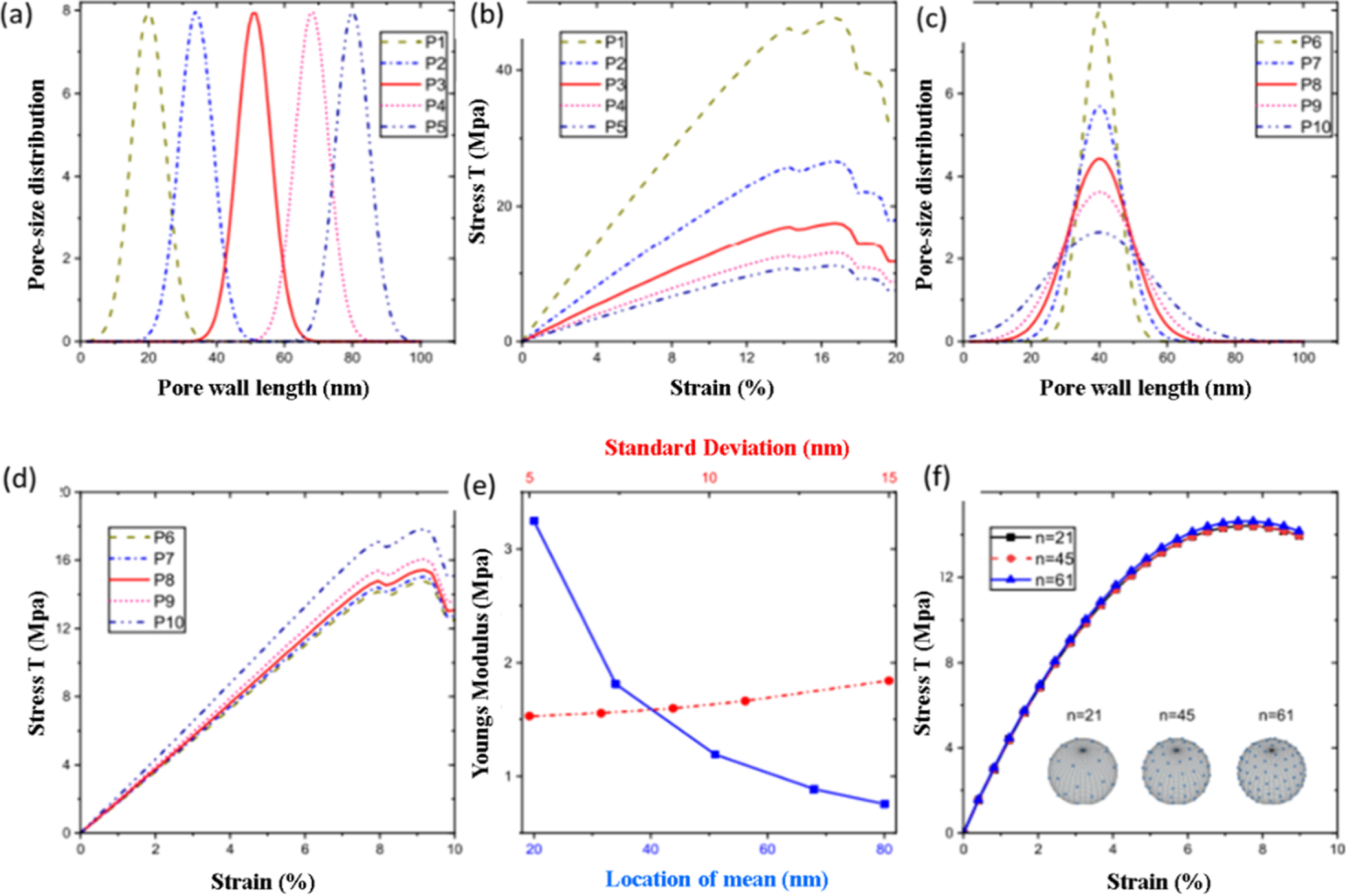
(a) Different pore size distributions considered based on the variation of the mean pore sizes, (b) influence of the mean pore size on the macroscopic tensile behavior, (c) different pore size distributions considered based on the variation of the standard deviation in the pore sizes, (d) influence of the standard deviation on the macroscopic tensile behavior, (e) Young’s modulus vs the mean and standard deviation of the pore size distribution, and (f) effect of the choice of numerical scheme within the microsphere model on the stress–strain behavior (the inset shows the three schemes, *n* = 21, 45, and 61). Reprinted with permission from Ameya, Influence of pore-size distributions and pore-wall mechanics on the mechanical behavior of cellular solids like aerogels, published by American Physical Society, 2021.^84^

The mechanical properties and flexibility of aerogels are strongly influenced by the shape, distribution, and size of their pores. Greater flexibility can result from larger, unevenly dispersed pores, as opposed to smaller, more evenly distributed pores. The capacity of aerogels to deform under force without breaking is significantly influenced by the pore size. Researchers Rege et al. investigated the effect of pore size distribution on the flexibility, as well as mechanical characteristics of nanoporous cellular materials such as aerogels. They suggested using a micromechanical model to develop an artificially normal pore size distribution and tested it to determine whether the macroscopic aerogel material was inelastic, elastic, or brittle. The pore size distribution spanning a range between 2 and 100 nm was selected to be described by a typical Gaussian distribution. According to the IUPAC definition;^81^ this includes mesopores, which are smaller than 2 nm, and macropores, which are larger than 50 nm. In their research, a normal distribution was considered while investigating the impact of pore diameters on an open-porous cellular material. As shown in Figure 12a, the mean size of the pores was previously believed to vary in several samples, ranging from 20 to 80 nm. Purely changing the mean pore size has a significant influence on the compressive performance as well as the macroscopic tensile for all other model parameters that remained constant. This impact on the stress–strain curve for the tensile materials is shown in Figure 12b. It demonstrated that how the constitutive behavior weakened as the mean pore size was increased and shifted to the right.^82^ The various pore size distributions are depicted in Figure 12c by simply modifying the standard deviation. The results for a specific mean are not particularly striking. The tensile stress–strain pattern for the various corresponding pore size distributions is shown in Figure 12d. The mechanical response is a little stiffer as the standard deviation became wider. Figure 12e, which showed a slight rise in Young’s modulus with widening standard deviation as recorded under compression, makes it more obvious. Recent research on carbon aerogels, however, has revealed that distinct mechanical properties can be seen in aerogels prepared with the same average density but with various pore diameters.^83^ Despite having comparable relative densities, carbon aerogels with smaller pores function more rigidly than those with larger pores. It would appear that further research is required to explore these size impacts, particularly when combined with the density ratio. The aforementioned results could vary if the pore wall thickness changes simultaneously. The material response is stiffer as the pore walls become thicker. The initial response further revealed a similar weakening under compression. For instance, the mechanical stiffness was decreased by a factor of more than one-third as the mean pore size was increased from 20 to 80 nm. The softening of the Young’s modulus as determined from the uniaxial compression models is shown by the blue curve in Figure 12e.

## Applications of Flexible Aerogles

**Figure 13:**
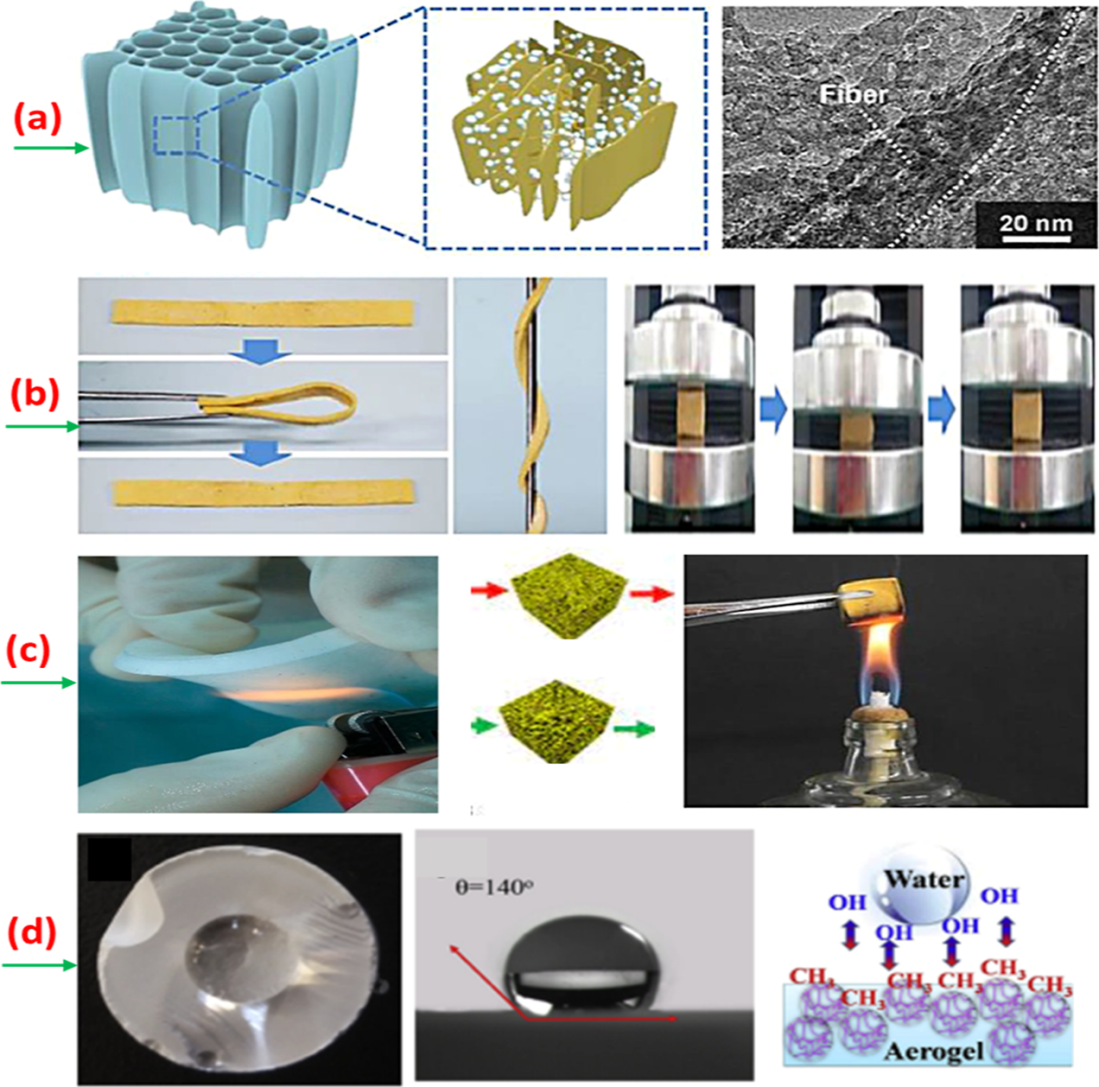
Polymer-based flexible aerogel (a) with an integrated double network, (b) use to make flexible and compressible strips, (c) use to develop flame-retardant sheets, and (d) use in superhydrophobic applications. Reprinted with permission from Jing Tian, Highly flexible and compressible polyimide/silica aerogels with integrated double network for thermal insulation and fire-retardancy, published by ELVISER, 2022.^87^

Flexible aerogels are unique materials with a wide range of outstanding physicochemical properties, such as mechanical, physical, and chemical properties. The most numerous applications of flexible aerogels include catalysis, thermal insulation, electrodes, solar thermal energy systems, oil spill cleaning, drug and protein delivery, medical implantable devices, and supercapacitors.^85^ Moreover, the most recent and cutting-edge technical uses of aerogels are in the field of biomedical engineering, energy systems, the environmental protection, and sensors.^86^Figure 13 shows the overview of different application fields of flexible aerogels.

### Flexible Aerogel for Biomedical Engineering

**Figure 14:**
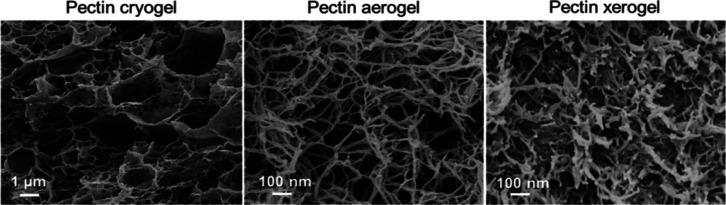
SEM micrographs of pectin cryogels, aerogels, and xerogels. Reprinted with permission from Sophie Groult, Pectin hydrogels, aerogels, cryogels, and xerogels: influence of drying on structural and release properties, published by ELVISER, 2021.^89^

Flexible aerogels have been extensively used in tissue engineering to regenerate a variety of tissues, including blood vessels, cartilage, bones, and skin. Muñoz-Ruíz et al. evaluated the aerogel made of collagen and alginate for the regeneration of various tissues using biobased materials to address the issue of potential autograft-related complications. They observed the effect of the supercritical drying process against porosity. The resultant alginate-collagen aerogel-based scaffold could be a platform for tissue engineering since it shows a highly interconnected network that aid in cell attachment.^88^Figure 14 shows that the drying method has a significant impact on the various properties of the pectin networks by using scanning electron microscopy.

### Use of Flexible Aerogels in the Field of Insulation and Energy Sensors

For a variety of thermal insulation-related applications, including vacuum insulation, cryogenic insulation, and building insulation, flexible aerogels are promising materials.^90^ These materials can be used for a variety of industrial and energy-related applications due to their high flexibility, transparency, low thermal conductivity, high surface area, and sharp pore size distribution.^91,92^ Aerogels are a good option for addressing serious environmental issues due to their low density and biodegradability.^93^ Recently, Han et al. utilized both freeze-casting and the thermal reduction method to produce polymeric flexible aerogels, where TiO_2_ and chitosan were used as thermal insulating materials. The fabricated aerogels showed high-temperature service performance, good thermal insulation, and excellent mechanical properties.^94^ Another potential application of flexible aerogels is in the field of energy and is being used in supercapacitors and batteries. Long et al. presented an original technique for the manufacture of N_2_-doped carbon aerogels for supercapacitors. They used glucose, cellulose nanofibers, and dicyandiamide as precursors to make the carbon aerogel from biomass. On flexible electronics, sensors, and energy storage/conversion devices, such as supercapacitors, the multifunctional materials were tested and found to have excellent thermal and mechanical properties.^95^

**Figure 15:**
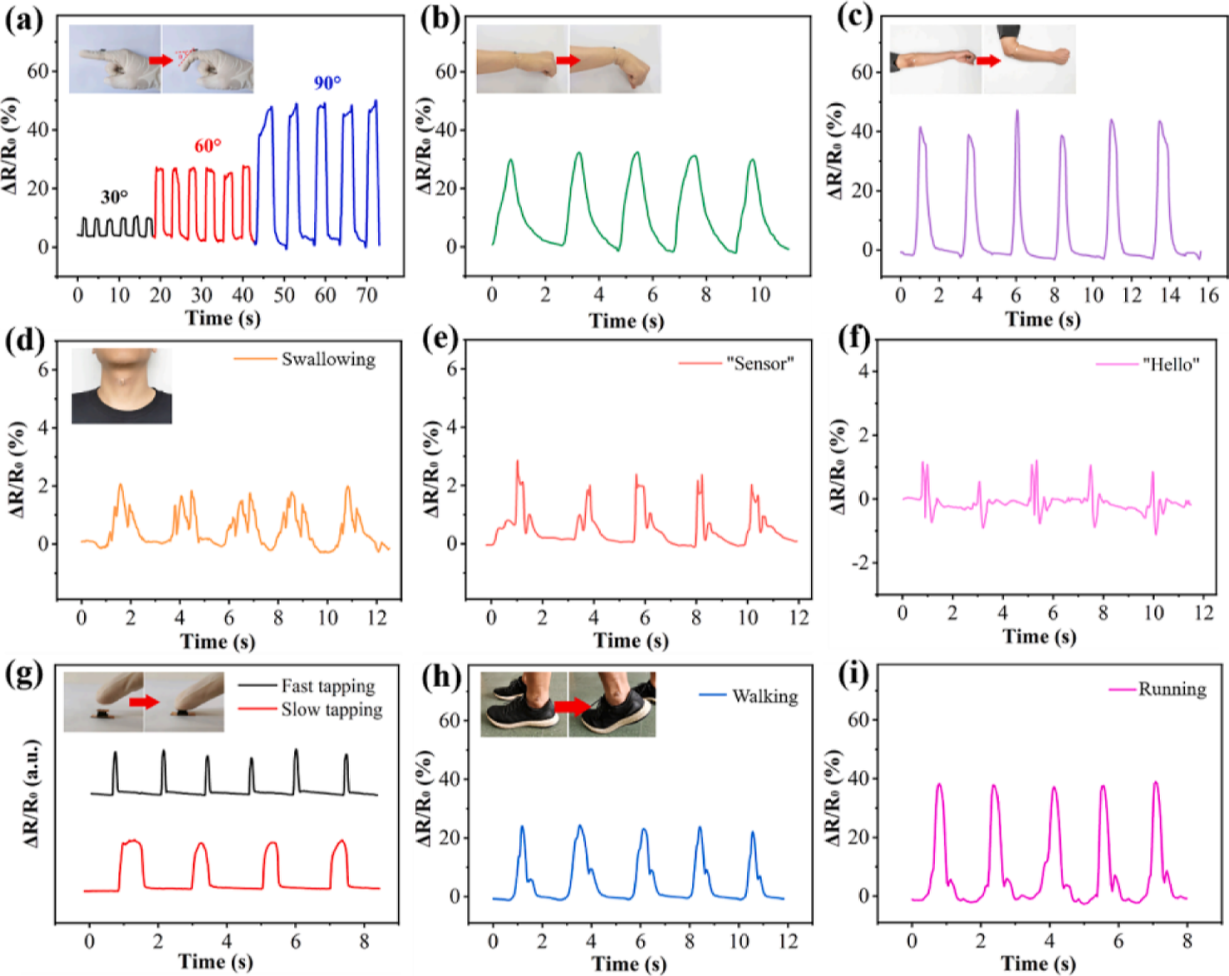
Detection in change of relative resistance of the sensor (a) applying bending movements of finger, (b) bending wrist at different angles, (c) bending elbow, (d) swallowing, (e) speaking “Sensor”, (f) speaking “Hello”, (g) tapping finger, (h) walking, and (i) running. [Zhipeng Yang], [Superhydrophobic MXene@carboxylated carbon nanotubes/carboxymethyl chitosan aerogel for piezoresistive pressure sensor]; published by [ELVISER], [2021].^96^

Yang et al. reported the development of electrically conductive and superhydrophobic aerogels by using a directional freeze-drying technique. The resultant aerogels presented potential applications in the field of piezoresistivity due to their microstructures resembling honeycombs. Moreover, the developed sensors have shown a wide range of detection, stability, excellent electrical repeatability, and quick response times. The made sensor was also used to measure how the human body moves, and it worked well even under humid or sweaty conditions. Additionally, the constructed sensor tracked finger joint movements in real time as shown in Figure 15.^96^

Alizadeh and Ahmadian fabricated a hydrogel with the combination of three-dimensional graphene aerogels by using an aqueous solution method. The resultant materials were used as an ammonia gas sensor and were able to rapidly detecting reversible smelling salts gas at encompassing temperature.^97^ Bi et al. reported the creation of carbon aerogel cluster electrodes mounted on a carbon ball. The primary objective of the proposed method is the creation of an electrochemical sensor from taros biomass. The proposed method demonstrated its potential as a powerful electrode for the fabrication of multifunctional electrochemical sensors for practical applications, and the results demonstrated a high electrochemical activity performance.^98^

### Use of Flexible Aerogels in the Building Industry

Because of their inherent characteristics of flexibility, high temperature insulation, and low thermal conductivity, the aerogels are potential alternative of traditional insulation materials.^99^ Recently, Aspen Aerogels, Inc., fabricated flexible, and transparent aerogels for daylighting purposes in new buildings with the trade name of Spaceloft. The product has a thermal conductivity of approximately 0.013 W/m·K, which is quite suitable for highly insulating aerogel windows. Recently, the fabrication of monolithic aerogel-based windows was proposed by the European Union project named HILIT. They combined vacuum glazing technology with 1–10 mbar pressure. The overall heat loss efficiency was about 0.66 W/m^2^·K, and the calculated solar transmittance *T*_SOL_ was more than 0.85. Furthermore, the heat loss coefficient was decreased with thickened aerogel glazing. Cabot Aerogel commercialized two aerogel products, Nanogel and Okagel. In 30 and 60 mm samples, Okagel has a thermal conductivity of 0.018 W/m·K and heat transmittance coefficients between 0.6 and 0.3 W/m^2^·K, respectively.^100^

## Conclusions

In summary, a brief review was compiled on hybrid and single-component flexible aerogels. This review describes the most promising way to overcome problems of nonflexible aerogels by introducing the novel flexible aerogels. In the case of introducing the flexibility, the advantages of introducing the organic part enhance the multifunctional properties (flexibility, insulation, and mechanical characteristics) in aerogels. The special class of flexible aerogels (single-component flexible aerogels) showed the tendency of higher mechanical properties along with excellent flexibility and thermal insulation. Moreover, it was analyzed that the most flexible structures can be produced by using the thermoplastic polymers. This demonstrates the recent interest and novelty of these materials in producing flexible aerogels. Furthermore, the supercritical drying method has an advantage over other drying techniques. During supercritical drying, the surface tensions in pores can be avoided, which in turn maintains the pore structure of aerogels. The detailed investigation of the chemical, mechanical, physical, and morphological properties of flexible aerogels has explored their use in wide range of technical applications such as biomedical engineering, insulation, energy sensor, and built-tech.
